# Crack-Based Composite Flexible Sensor with Superhydrophobicity to Detect Strain and Vibration

**DOI:** 10.3390/polym16172535

**Published:** 2024-09-07

**Authors:** Yazhou Zhang, Huansheng Wu, Linpeng Liu, Yang Yang, Changchao Zhang, Ji’an Duan

**Affiliations:** 1 State Key Laboratory of Precision Manufacturing for Extreme Service Performance, College of Mechanical and Electrical Engineering, Central South University, Changsha 410083, China; asiazhang@126.com (Y.Z.); 233812052@csu.edu.cn (H.W.); linpengliu@csu.edu.cn (L.L.); duanjian@csu.edu.cn (J.D.); 2 China Railway 14 Bureau Group Co., Ltd., Jinan 250101, China; 3 Key Laboratory of Bionic Engineering, Ministry of Education, Jilin University, Changchun 130022, China; changchaozhang@jlu.edu.cn

**Keywords:** flexible, sensor, easy fabrication, superhydrophobic, vibration monitoring

## Abstract

Vibration sensors are widely applied in the detection of faults and analysis of operational states in engineering machinery and equipment. However, commercial vibration sensors with a feature of high hardness hinder their usage in some practical applications where the measured objects have irregular surfaces that are difficult to install. Moreover, as the operating environments of machinery become increasingly complex, there is a growing demand for sensors capable of working in wet and humid conditions. Here, we present a flexible, superhydrophobic vibration sensor with parallel microcracks. The sensor is fabricated using a femtosecond laser direct writing ablation strategy to create the parallel cracks on a PDMS film, followed by spray-coating with a conductive ink composed of MWCNTs, CB, and PDMS. The results demonstrate that the developed flexible sensor exhibits a high-frequency response of up to 2000 Hz, a high acceleration response of up to 100 m/s^2^, a water contact angle as high as 159.61°, and a linearity of 0.9812 between the voltage signal and acceleration. The results indicate that the sensor can be employed for underwater vibration, sound recognition, and vibration monitoring in fields such as shield cutters, holding significant potential for mechanical equipment vibration monitoring and speech-based human–machine interaction.

## 1. Introduction

Currently, flexible sensors have garnered significant attention from researchers due to their extensive applications in industrial production, agriculture, medicine, military, and environmental fields [1,2,3,4,5,6]. The movement of objects or the operation of mechanical equipment invariably generates vibrations. Vibration signals are one of the primary channels through which humans and natural organisms acquire information from their environment [7]. Consequently, vibration sensors are widely used in mechanical equipment, transportation, building facilities, and biomedical electronic devices to capture the vibration signals of the monitored objects, thereby enabling state monitoring and fault analysis [8,9,10,11,12,13]. Vibration sensors can convert the mechanical vibration signals of the monitored object into electrical signals. By analyzing the frequency, acceleration, and amplitude characteristics of these electrical signals, we can monitor and evaluate the operating status of mechanical equipment, human health conditions, and the status of building facilities and bridges [14,15,16,17,18,19]. This enables us to diagnose faults or diseases, facilitating early prevention and intervention.

Traditional vibration sensors are predominantly made from materials such as metals, ceramics, silicones, and sapphires, and their fabrication involves complex and high-cost techniques like photolithography, ion implantation, and electron beam evaporation [20,21,22,23,24,25,26]. Furthermore, rigid vibration sensors made from these materials have high installation requirements and cannot be stably mounted on the irregular surfaces of critical mechanical equipment, severely limiting their application on irregular surface objects. There have also been many studies using PDMS flexible polymers to fabricate flexible sensors, spraying conductive materials on the surface of PDMS polymers, or using inverted molding methods to create surface structures for enhanced sensing performance. However, these reported studies are unable to respond to high-frequency vibrations and do not have the superhydrophobic properties to work stably in wet or underwater environments [27,28,29]. The operational environments for mechanical and transportation equipment are often complex, frequently requiring functionality in rainy or humid conditions. This presents a significant challenge for the stable detection capabilities of vibration sensors. Therefore, developing flexible, cost-effective vibration sensors that can reliably detect vibrations in humid environments is of great importance for enhancing the monitoring accuracy of mechanical equipment and expanding their range of applications [30,31,32,33,34,35,36,37].

With the rapid development of artificial intelligence (AI) and the Internet of Things (IoT), many flexible sensors capable of detecting vibration stimuli have been developed [38,39,40,41]. For instance, Zou et al. created a flexible, adaptive triboelectric vibration sensor based on conductive sponge-silicone, which exhibited excellent flexibility and could detect vibration frequencies ranging from 10 Hz to 100 Hz [1]. Wang et al. developed a crack-based flexible sensor inspired by scorpion sensory organs, capable of detecting vibrations up to 103 Hz [42]. While many flexible sensors are now capable of converting vibration stimuli into electrical signals, most of them fail to respond to vibration stimuli with frequencies as high as several hundred hertz due to the viscoelasticity of flexible materials. Additionally, stable detection of vibration stimuli in wet environments poses a significant challenge for flexible sensors. Therefore, there is an increasingly urgent need to design flexible sensors that can detect a wide frequency response range and stably detect vibration stimuli underwater [43,44,45].

In this study, we developed a highly sensitive superhydrophobic flexible vibration sensor based on the strain effect. The sensor uses a flexible polymer material in the conductive sensing layer and parallel penetrating slits in the polymer-sensitive membrane designed and fabricated by femtosecond laser ablation, which improves the sensitivity to weak vibrations and broadens the response frequency and acceleration ranges, as well as the stability in humid environments. By using femtosecond laser ablation to fabricate parallel penetrating slits on the polymer-sensitive membrane, we realized the conversion of mechanical deformation to resistive signals, and thus output the corresponding electrical signals under vibration. This approach not only improves the performance of the sensor but also expands the application of polymer materials in flexible vibration sensing. The main objective of this paper is to present the design, fabrication, and characterization of this innovative flexible vibration sensor based on PDMS polymer. We emphasize its potential applications in areas such as real-time monitoring, speech recognition, and vibration detection in the complex environment of shield cutter boring operations. This study emphasizes the importance of designing and fabricating structures on the sensing layer of polymeric materials in improving the performance of the sensor and broadening its applications in engineering and technology.

## 2. Materials and Methods

### 2.1. Materials

PDMS (Sylgard 184) was purchased from Dow Corning Corp. MWCNTs (average diameter 10–20 nm, average length 10–30 μm) were purchased from Nanjing XFNANO Materials Tech. Co., Ltd., Nanjing, China. Ethyl acetate was purchased from Shanghai Sinopharm Chemical Reagent Co., Ltd., Shanghai, China. CBs (ECP-600JD) were purchased from Tianjin Aiweixin Chemical Technology Co., Ltd., Tianjin, China.

### 2.2. Preparation of Conductive Ink

Quantities of 3.3 g of PDMS (10:1 ratio of reagent A to reagent B), 50 g of ethyl acetate, 0.1 g of MWCNTs, and 0.5 g of CB were mixed, followed by magnetic stirring at 400 rpm for 0.5 h at room temperature. Whereafter, an ultrasonic processing was needed and kept for 10 min. Finally, the conductive ink was prepared.

### 2.3. Preparation of Vibration Sensor

First, we mixed the PDMS prepolymer and curing agent in a weight ratio of 10:1. Then, we dried the mixture at 70 °C for 60 min to obtain fully cured PDMS film. Then, a femtosecond laser (HR-Platform-0203, Wuhan Huaray Precision Laser Co., Ltd., Wuhan, China) with a power percentage of 150 kHz, scanning speed of 500 mm/s, and repetition rate of 50 was used to process parallel through-hole structures on the PDMS film surface, totaling four lines. Finally, we cut the PDMS into a size of 30 mm × 10 mm. The processed and cut PDMS was then cleaned with anhydrous ethanol in an ultrasonic bath for 10 min to remove the dust generated by the femtosecond laser ablation. Subsequently, it was dried at 70 °C for 10 min until the anhydrous ethanol completely evaporated. The prepared conductive ink was uniformly sprayed onto the structured PDMS film surface using a spray gun. After spraying, the samples were placed in a drying oven at 100 °C for 1 h to ensure complete evaporation of the ethyl acetate in the conductive ink and complete curing of the PDMS. The conductive silver paste was evenly coated at both ends of the conductive layer, and copper foil was attached to the conductive silver paste to serve as electrodes. The samples were then placed in an oven at 120 °C and heated for 30 min until the conductive silver paste was fully sintered. Finally, the vibration sensor fabrication was completed.

### 2.4. Characterization

The vibration signals of varying frequencies, waveforms, and accelerations were generated by the excitation system, comprising a vibration exciter (SA-JZ002, Wuxi Shiao Tech. Ltd., Wuxi, China), power amplifier (SP-PA003, Wuxi Shiao Tech. Ltd., China), signal generator (DG1022Z, RIGOL, Beijing, China), dynamic signal analyzer (SA1808A2, Shiao, China), and commercial accelerometer (SACL001ZKE, Wuxi Shiao Tech. Ltd., China). The contact angle of the strain sensor under different cycles and different liquids was measured using an optical contact angle meter (Harke, Beijing, China). The resistance change signal of the sensor’s tensile strain was collected by a digital multimeter (DAQ6510, KEITHLEY, Cleveland, OH, USA).

## 3. Results and Discussion

### 3.1. Design of the Crack-Based Composite Flexible Sensor

**Figure 1:**
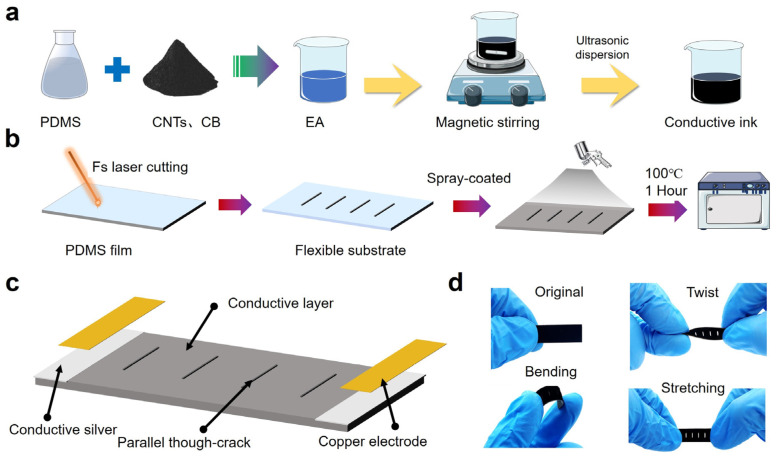
Preparation of the conductive ink and flexible vibration sensor. (**a**) Fabrication process of the conductive ink composed of PDMS, CNTs, CBs, and ethyl acetate. (**b**) Structural and conductive layer fabrication of the vibration sensor. (**c**) Schematic diagram showing the architecture of the vibration sensor. (**d**) Optical images of the vibration sensor under different mechanical loads, showing the flexibility of the sensor.

Figure 1a illustrates the composition and preparation process of the conductive ink, which comprised CNTs, CBs (carbon-based materials with excellent conductivity), and PDMS. PDMS composed of two-component materials has a high viscosity and can be used as an adhesion agent and serve as a substrate matrix to allow conductive materials to be embedded. To form conductive ink, ethyl acetate was selected as the organic solvent because it could not only reduce the high viscosity of PDMS but also disperse the carbon nanotubes or nanoparticles. First, the PDMS base agent, CNTs, CB, and ethyl acetate were mixed in a specific ratio, followed by a magnetic stirring and ultrasonic oscillating process, respectively, to make sure the conductive materials were uniformly dispersed in the solvent. Before spraying the conductive ink, the other component, the PDMS curing agent, was added to the solution with a ratio of 1:10 to the base agent, then magnetic stirring was used on the solution for a few minutes.

Figure 1b illustrates the fabrication process of the sensitive membrane by femtosecond laser and the conductive coating by a spray-coated method. First, a PDMS film was prepared with a thickness of 0.4 mm, a length of 30 mm, and a width of 10 mm. Then, the femtosecond laser was used to ablate the PDMS film to form parallel, through cracks. There were four parallel, through cracks on the PDMS film with an interval of 4 mm, and the length of each crack was set as 4 mm. The processed PDMS film after the femtosecond laser was cleaned by using anhydrous ethanol to remove the remained materials after ablation so that the spray-coated layer could achieve a better interfacial bonding with the PDMS substrate. After the PDMS substrate was spray-coated with PDMS/CNTs/CBs/ethyl acetate conductive ink, the sample was then heated in a drying oven at 100 °C for one hour, to make the conductive ink form a stable layer. During the heating process, ethyl acetate completely evaporated due to its boiling temperature being only 77.2 °C. Figure 1c presents the architecture of the vibration sensor, composed of a conductive layer with parallel through cracks, conductive silver paste layer, and copper electrodes. Figure 1d shows the deformation of the vibration sensor under different external loads, demonstrating the sensor’s flexibility in response to tension, bending, and torsion.

### 3.2. Working Mechanism of the Crack-Based Composite Flexible Sensor

**Figure 2:**
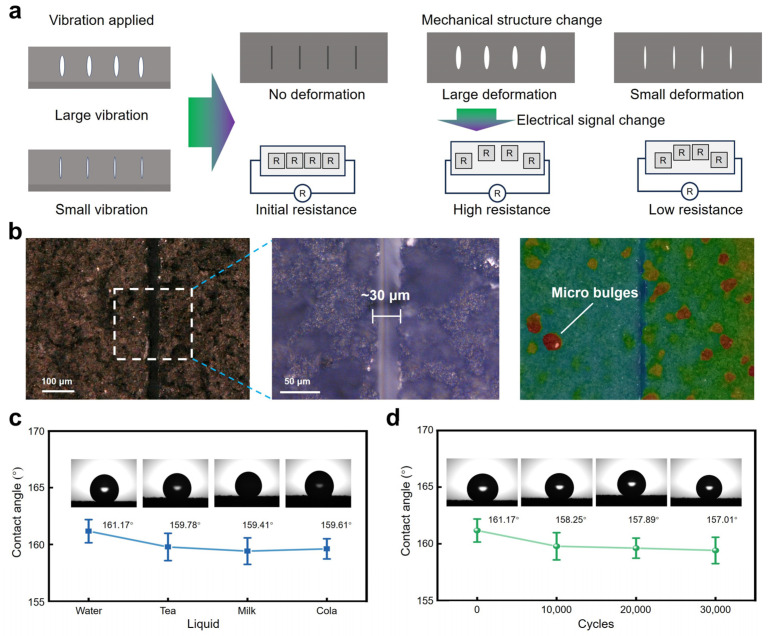
The sensing mechanism and optical surface images of the sensor, as well as the hydrophobic performance. (**a**) Sensing mechanism of the sensor under vibrations. (**b**) Optical images of the sensor’s surface obtained from an ultra-depth three-dimensional microscope. (**c**) Contact angles of the sensor when different liquids (water, tea, milk, and cola) drop on the sensor’s surface. (**d**) Water contact angles of the sensor after being subjected to different vibration cycles.

Figure 2a shows the working principle of the sensor under vibrations. The flexible sensor had great deformation when it suffered from a large vibration since the widths of the cracks had obvious changes when compared with those of the sensor under a small vibration. Changes in the vibration frequency or acceleration caused alterations in the mechanical structure of the vibration sensor, consequently leading to corresponding changes in the electrical signal output by the sensor. When the sensor received external vibration stimuli, the conductive sensing layer underwent vertical oscillations accordingly. When the sensor was at rest, the conductive sensing layer remained unchanged structurally, maintaining its initial resistance value. However, during low-frequency or high-acceleration external vibrations, the impact on the conductive sensing layer resulted in significant mechanical deformation. This widened the through-slit structures, causing substantial overall deformation in the conductive sensing layer and significant changes in resistance. Conversely, during high-frequency or low-acceleration vibrations, the vertical oscillations of the conductive sensing layer were minimal, resulting in slight deformations and negligible changes in resistance. Thanks to the sensor’s excellent flexibility, stretchability, and parallel distribution of through cracks, it could effectively respond to even subtle vibration stimuli with corresponding electrical signal outputs.

Figure 2b shows the surface geometry of the sensor characterized by an ultra-depth three-dimensional microscope. Linear-shaped cracks can be clearly observed whose width is relatively uniform at ~30 μm. After the conductive ink was spray-coated on the surface of the PDMS substrate, irregular fine protrusions were formed on the conductive layer due to a general atomization effect of the spray head. However, the undesired irregular fine protrusions offered the sensor an unexpected superhydrophobic function. It is known that PDMS is hydrophobic [46,47]. When carbon-based materials were added to the PDMS matrix, irregular fine protrusions could be formed after the carbon/PDMS conductive ink spray-coated on the sensor’s surface, changing the hydrophobic surface of the pure PDMS surface into a superhydrophobic surface of hybrid conductive coating. Figure 2c shows a water contact angle of 161.17° of the hybrid conductive coating surface, which is much higher than the defined superhydrophobic angle of 150°.

The superhydrophobicity of the sensor’s conductive sensing layer determined its versatility in various working conditions, while most sensors whose surfaces use conductive coatings as sensing layers lack the ability to work in wet or humid environments [48,49,50,51,52,53]. As shown in Figure 2c, four common liquids (tea, milk, water, and cola) were selected to assess the hydrophobic performance of the sensor. Results indicated that the contact angles of the sensor surface towards different liquid droplets were all greater than 150°, demonstrating excellent applicability of the surface sensor in everyday humid environments. Figure 2d demonstrates the water contact angle of the sensor under different cycles of vibration at a frequency of 100 Hz and an acceleration of 10 g. Results indicated that the conductive sensing layer maintained its superhydrophobic properties even after 40,000 cycles, showing features such as high reliability and mechanical stability.

### 3.3. Sensing Performance of the Crack-Based Composite Flexible Sensor

**Figure 3:**
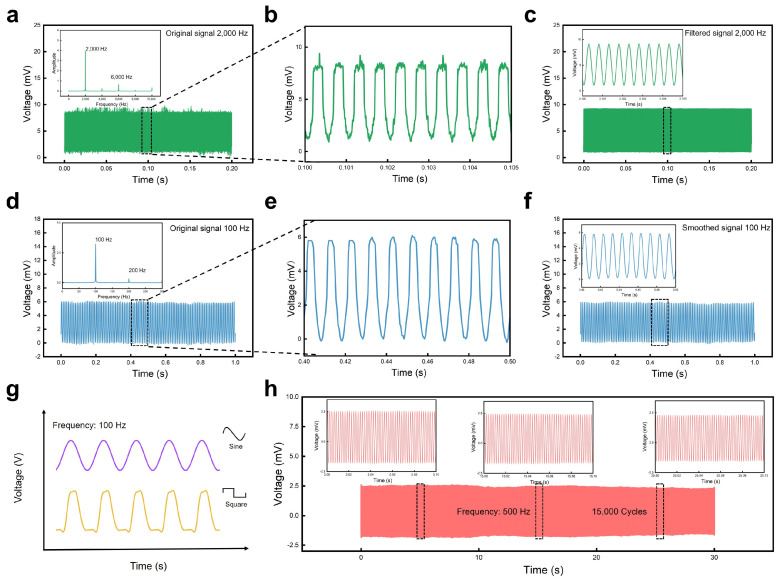
The response of the flexible sensor to vibrations. (**a**) Original signals from the sensor under an applied periodic vibration with a frequency of 2000 Hz. Inset: FFT analysis of the original signals, showing a dominant frequency of 2000 Hz. (**b**) Amplified view of the signal from 0.1 s to 0.105 s in (**a**). (**c**) Voltage change of the sensor over time after filtering noise from the original signal in (**a**). (**d**) Original signals from the sensor under an applied periodic vibration with a frequency of 100 Hz. Inset: FFT analysis of the original signals, showing a dominant frequency of 100 Hz. (**e**) Amplified view of the signal from 0.40 s to 0.50 s in (**d**). (**f**) Voltage change of the sensor over time after smoothing from the original signal in (**d**). (**g**) Real-time response of the sensor to vibrations with different waveforms at a frequency of 100 Hz. (**h**) Signal output of the sensor under an applied periodic vibration with a frequency of 500 Hz, recording the sensor’s responses of 15,000 cycles within a duration of 30 s. Insets: partially magnified curves for different time stages.

After conducting the basic characterization of the mechanical structure and sensing mechanism of the vibration sensor, the sensor’s performance was tested and analyzed, particularly its vibration frequency response. A testing system mainly consisting of a signal generator, power amplifier, signal acquisition device, shaker, and power supply was used to evaluate its fundamental vibration response performance. The sensor was connected in series with a 1 kΩ resistor. A 5 V voltage powered the circuit so that the resistance change of the sensor under applied vibration turned to voltage changes when we measured the separate voltage of the sensor with high-frequency sampling (128 K Sa/s). The ability of a vibration sensor to respond to high-frequency vibrations is a crucial metric for evaluating its responsiveness, as it signifies the sensor’s finer sensing capability. To assess the sensor’s response to high-frequency vibrations, the vibration frequency of the shaker was set to 2000 Hz. As depicted in Figure 3a, the sensor exhibited stable responses to vibrations with frequencies as high as 2000 Hz, according to the fast Fourier transform analysis result of the output signals whose main frequency was 2000 Hz, aligning perfectly with the input vibration signal. Figure 3b displays a magnified view of the output signal ranging from 0.1 s to 0.105 s, showing the sensor’s stable responses to periodic vibration stimuli. Figure 3c depicts the signal after low-pass filtering to remove noise above 2100 Hz, which closely matched the input vibration shock signal received by the sensor.

Following the same procedure as the high-frequency vibration response test, we set the vibration frequency of the shaker to 100 Hz to assess the sensor’s low-frequency response capability. Figure 3d shows the original electrical signal output from the sensor and the inset in Figure 3d reveals a dominant frequency of 100 Hz which is highly consistent with the input vibration frequency. Figure 3e provides a magnified view of time ranging from 0.40 s to 0.50 s of the signal in Figure 3d, showing that the sensor has more regular and stable waveform responses to vibrations with low frequencies when compared to the responses to vibrations with high frequencies. Figure 3f depicts the plot after smoothing the raw electrical signal; more stable and regular waveforms without signal burrs can be observed in the inset. Therefore, the developed vibration sensor exhibited excellent response capabilities to both low-frequency and high-frequency vibrations. Further investigation into the sensor’s low-frequency response performance involved outputting sine waveform and square waveforms of vibrations separately at a frequency of 100 Hz. As shown in Figure 3g, the response signal of the vibration sensor varied accordingly with these two waveforms, indicating its capability to respond to different vibration signal types. This finding holds significant practical application prospects in areas such as mechanical fault detection and signal classification.

The lifespan of a vibration sensor is a significant factor limiting its application, as excellent sensors must demonstrate the ability to operate over extended periods. To assess this, we secured the sensor to the shaker and subjected it to continuous vibration shocks at a frequency of 500 Hz for 30 s, totaling 15,000 shock cycles. As depicted in Figure 3h, the sensor continued to stably output signals throughout this duration. This indicated that the vibration sensor possessed outstanding longevity, enabling prolonged vibration detection capabilities.

**Figure 4:**
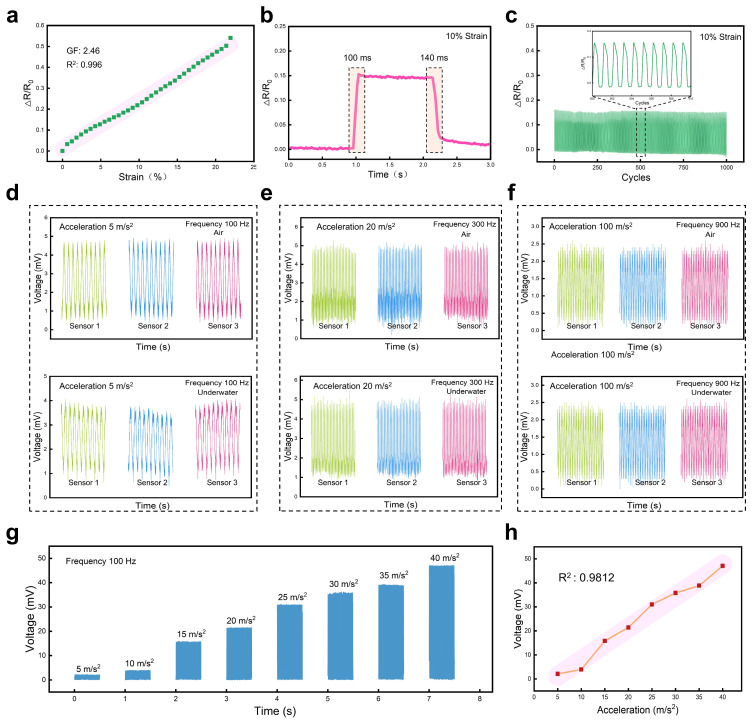
The response of the vibration sensor to tensile strain and underwater vibration. (**a**) Sensitivity and linearity of the sensor to tensile strain. (**b**) Response/recover time of the sensor at 10% strain. (**c**) Signal output of the sensor for 1000 cycles to the tensile strain of 10%. (**d**) Electrical response of the three sensors to vibration with a frequency of 100 Hz and an acceleration of 5 m/s^2^ in air and underwater, respectively. (**e**) Electrical response of the three sensors to vibration with a frequency of 300 Hz and an acceleration of 20 m/s^2^ in air and underwater, respectively. (**f**) Electrical response of the three sensors to vibration with a frequency of 900 Hz and an acceleration of 100 m/s^2^ in air and underwater, respectively. (**g**) Relative voltage changes of the sensor under vibrations at a fixed frequency of 100 Hz but different accelerations at 5, 10, 15, 20, 25, 30, 35, and 40 m/s^2^, respectively. (**h**) Relationship between the sensor’s voltage response and acceleration under vibrations at a fixed frequency of 100 Hz.

Due to the sensor’s flexibility and stretchability, the sensor could also be used as a strain sensor. Figure 4a shows the relative resistance change of the sensor over applied strains, demonstrating that the sensor had a gauge factor of 2.46 during the working range of 0~22% strain, and a dependent coefficient of 0.996 (R^2^) could be obtained after fitting, exhibiting higher linearity to strain when compared to other reported flexible strain sensors [54,55,56,57,58]. Figure 4b displays the sensor’s response/recovery time to a tensile strain of 10%, with a response time of ~100 ms and a recovery time of ~140 ms. We also tested the sensor’s stability under tensile cycles. As shown in Figure 4c, when the sensor was subjected to an applied tensile strain of 10%, the sensor’s responses behaved regularly and stably without obvious resistance drift during 1000 cycles.

Since the sensor’s conductive sensing layer had a superhydrophobic property, further testing was conducted to evaluate the sensor’s performance underwater. In addition, to verify that the sensors had reliable reproducibility, three sensors manufactured in the same process were selected to test vibration shocks at different frequencies and accelerations. Different frequencies and accelerations of vibration shocks were selected for testing. The sensor was placed in air and underwater, respectively, to test its responses under vibrations with different frequencies and accelerations. As shown in Figure 4d, the sensor worked normally in both air and underwater when vibration with a frequency of 100 Hz and an acceleration of 5 m/s^2^ were applied to the sensor, and the vibration of the three sensors in the same environment could output electrical signals with the same amplitude, indicating that the sensors had a reliable reproducibility. Figure 4e,f show the resistance change of the sensor when it worked in both air and underwater at different frequencies and accelerations. In comparison, the sensor’s response in the air behaved more stably and regularly than that underwater. Also, the maximum voltage amplitude of the sensor in the air was slightly larger than the peaks generated underwater. It may be the resistance of water that weakened the mechanical deformation so that the sensor’s resistance varied less. However, the test results in the air or underwater demonstrated the sensor could work in wet environmental conditions due to its great superhydrophobicity. Figure 4g shows the sensor’s voltage responses to vibrations with different accelerations but a fixed frequency of 100 Hz. When the applied acceleration ranged from 5 m/s^2^ to 40 m/s^2^ with an interval of 5 m/s^2^, the sensor exhibited significant distinguishability in response to different accelerations. We counted the corresponding voltage peak of each acceleration and then plotted it in Figure 4h; a correlation coefficient of 0.9812 (R^2^) was obtained, showing that the sensor had a potential resolution to different accelerations.

### 3.4. Application of the Crack-Based Composite Flexible Sensor

**Figure 5:**
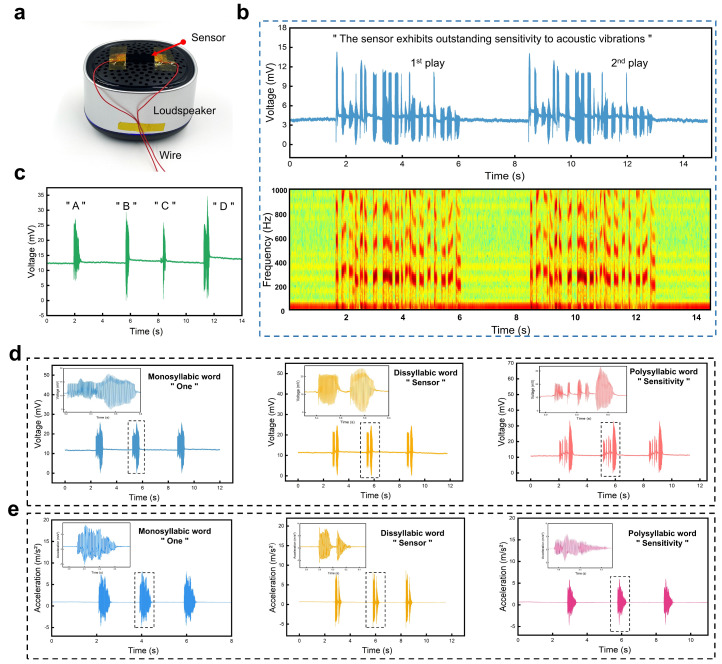
Vibration monitoring and speech recognition performance of the developed sensor. (**a**) Optical image of the flexible sensor installed on the surface of a speaker. (**b**) Output response signals of the sensor when the speaker played a sentence twice and the spectrogram analysis for the recorded electric signals. (**c**) Voltage responses of the sensor to vibrations generated from the speaker which played four letters in turn. (**d**) Voltage responses of the vibration sensor to three utterances, including the monosyllabic word “one”, the disyllabic word “sensor”, and the polysyllabic word “sensitivity”. (**e**) Voltage responses of the commercial accelerometers to three utterances, including the monosyllabic word “one”, the disyllabic word “sensor”, and the polysyllabic word “sensitivity”.

Dynamic and continuous mechanical vibrations are widely present in everyday life. However, detecting such subtle dynamic vibration shocks poses a significant challenge for flexible sensors. To characterize the developed sensor’s detection of dynamic mechanical vibrations, we constructed an experimental setup, as shown in Figure 5a. The sensor was horizontally placed on a speaker to enable real-time detection of vibrations on the speaker’s surface. We used a computer to control the speaker to play the “The sensor exhibits outstanding sensitivity to acoustic vibrations” sentence twice, the output results of the vibration sensor are depicted in Figure 5b. They indicated a high degree of consistency between the vibration waveforms generated before and after playing the sentence twice, demonstrating the excellent speech recognition capability of the vibration sensor. The voltage signal output by the vibration sensor was processed by a short-time Fourier transform (STFT) to obtain the corresponding spectral diagram and reveal the voice print of the captured signal. Obviously, the two output electrical signals showed good repeatability in the time, frequency, and amplitude domains. Figure 5c presents the sensor’s responses recorded when the speaker played the letters A, B, C, and D in turn, demonstrating the vibration sensor’s outstanding detection and recognition capability of the instantaneous sound wave vibrations. Interestingly, the vibration sensor also exhibited the ability to detect and distinguish words with different syllables. The speaker cycled the monosyllabic word “one”, the two-syllabic word “sensor”, and the multi-syllabic word “sensitivity” three times each, and the vibration sensor and the commercial accelerometer sensor response output results are shown in Figure 5d,e. The results show that the vibration sensor output electrical signal waveforms that were consistent with the number of syllables in the word and had excellent speech recognition capability, whereas, since the commercial accelerometer was prepared from a rigid material, it could not be in conformal contact with the object under test and therefore was unable to recognize multi-syllable words. Therefore, flexible vibration sensors with excellent flexibility and superior speech recognition ability are expected to be widely used in human–computer interaction scenarios involving voice control.

Based on the previous experimental results, the developed flexible sensor not only exhibited excellent detection capabilities for high-frequency vibrations but also showed good detection capabilities for low-frequency vibrations. Apart from testing the sensor’s performance to respond to sound, we also measured the sensor’s response to vibrations with a low frequency that are typically generated during the operation of large machinery. Shield machines are crucial mechanical equipment for transportation infrastructure construction, and the vibration signals at the interface between the cutter head and the rock can reflect its operational status and fault information. Therefore, detecting the vibration signals during the operation of the shield machine cutter head can further elucidate the coupling mechanism between the cutter head and the rock and evaluate its operational status.

**Figure 6:**
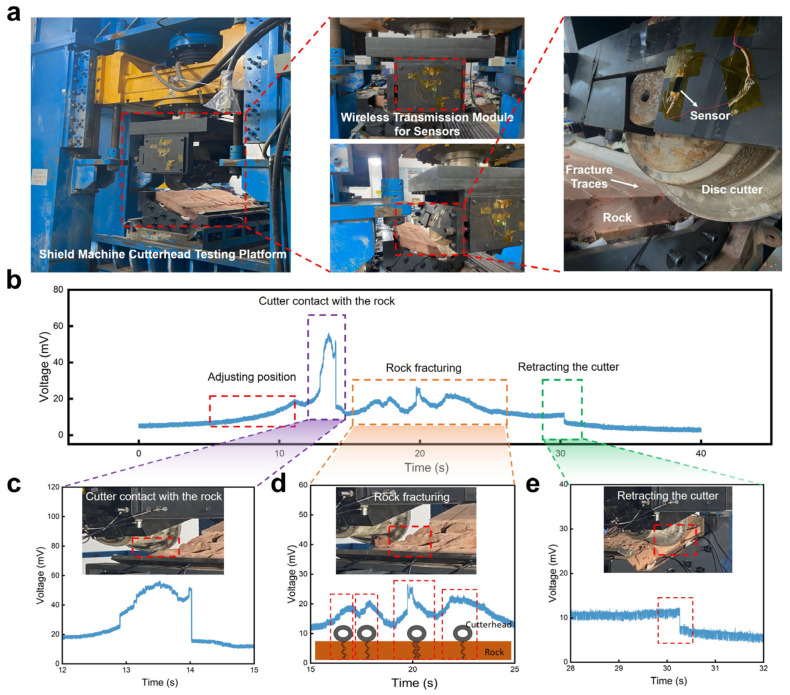
Vibration monitoring and analysis for the shield machine cutter head when fracturing a rock. (**a**) Optical image of the shield machine cutter head’s testing platform and the installation of the vibration monitoring system. (**b**) Real-time voltage signals output from the flexible vibration sensor during the rock fracturing process. (**c**) An enlarged view from (**b**) at the stage when the disc cutter contacts with the rock. (**d**) An enlarged view from (**b**) at the stage when the disc cutter begins to fracture the rock. Each peak shows the positions where the rock is cracked thoroughly. (**e**) An enlarged view from (**b**) at the stage when the disc cutter is retracted.

As shown in Figure 6a, the shield machine cutter head rock-fracturing test platform mainly consisted of the cutter base, disc cutter, rock, and hydraulic lifting device. The developed sensor was stuck on the base where the disc cutter was installed, to monitor the vibration signals generated during the rock fracturing process in real time. The vibration sensor converted the mechanical vibration signals generated by the disc cutter’s cut rock into electrical signals, which were then collected by a wireless transmission device and transmitted to a computer instantaneously. The signals collected during a complete working cycle of the cutter head are shown in Figure 6b, indicating that vibration sensors could monitor the mechanical vibration signals of the shield machine cutter head in different states. A further analysis of the collected vibration signals allowed us to identify four operational conditions: the adjustment of the cutter head, the contact between the cutter head and the rock, the fracturing of the rock, and cutter retraction. As shown in Figure 6c, the cutter head was driven by the hydraulic lift to adjust the relative position of the cutter head and the rock, so that the sensor output a relatively slow voltage signal. When the disc cutter was in contact with the rock, a large shock vibration was generated, which made the sensor output a sudden increased voltage signal. The vibration sensor could output electrical signals in response to different degrees of rock breakage, as shown in Figure 6d. During the process of rock crushing, the rock was cracked four times at different positions. According to the amplitude of the sensor output voltage peak signal, the degree of rock crushing could be reflected. Obviously, the crack generated in the third rock crushing was the largest, so the corresponding voltage peak value was the highest, reaching 25 mv. Similar to the contact between the cutter head and the rock, when the cutter head left the rock, it also produced an instantaneous shock vibration, but its vibration amplitude was small, as shown in Figure 6e. Therefore, the developed vibration sensors possessed excellent capabilities for detecting low-frequency vibrations generated during the process of shield machine cutter head rock fracturing, enabling the identification of different operational conditions.

## 4. Conclusions

In this work, we developed a flexible vibration sensor utilizing spraying and femtosecond laser technology that exhibited excellent capability in detecting strain and vibration, as well as superhydrophobic properties. The innovative design of parallel penetrating crack structures on the polymer sensing layer significantly improved its electromechanical signal conversion efficiency. Experimental results showed that the sensor was capable of detecting vibrations with frequencies of up to 2000 Hz, including those in underwater environments, highlighting its robustness and versatility. In addition, the sensor’s ability to accurately detect and recognize voice vibration, as well as monitor vibration in the cutter plate of a shield tunneling machine, highlights its potential for practical application in the assessment of machinery operating conditions. The simplicity and cost-effectiveness of its fabrication process further add to its appeal, making it a promising candidate for widespread use in machinery vibration monitoring and human–computer interaction. This study not only introduced an advanced method of using polymer-based materials to improve the performance of vibration sensors but also broadened the application of flexible sensors in challenging environments. The results of the study indicate a great potential for future developments in the fields of mechanical system monitoring and smart interface technologies, where reliable and high-performance vibration sensors are essential.

## Data Availability

The original contributions presented in the study are included in the article; further inquiries can be directed to the corresponding author.
